# Reversible Audiometric Threshold Changes in Children with Uncomplicated Malaria

**DOI:** 10.1155/2013/360540

**Published:** 2013-03-07

**Authors:** George O. Adjei, Bamenla Q. Goka, Emmanuel Kitcher, Onike P. Rodrigues, Ebenezer Badoe, Jorgen A. L. Kurtzhals

**Affiliations:** ^1^Centre for Tropical Clinical Pharmacology and Therapeutics, College of Health Sciences, University of Ghana Medical School, P.O. Box 4236, Korle Bu, Accra, Ghana; ^2^Department of Child Health, University of Ghana Medical School, P.O. Box 4236, Korle Bu, Accra, Ghana; ^3^Ear, Nose and Throat Unit of Department of Surgery, University of Ghana Medical School, P.O. Box 4236, Korle Bu, Accra, Ghana; ^4^Centre for Medical Parasitology at Department of Clinical Microbiology, Copenhagen University Hospital (Rigshospitalet) and Department of International Health, Immunology and Microbiology, University of Copenhagen, Denmark

## Abstract

*Background*. *Plasmodium falciparum* malaria, as well as certain antimalarial drugs, is associated with hearing impairment in adults. There is little information, however, on the extent, if any, of this effect in children, and the evidence linking artemisinin combination therapies (ACTs) with hearing is inconclusive. *Methods*. Audiometry was conducted in children with uncomplicated malaria treated with artesunate-amodiaquine (*n* = 37), artemether-lumefantrine (*n* = 35), or amodiaquine (*n* = 8) in Accra, Ghana. Audiometry was repeated 3, 7, and 28 days later and after 9 months. Audiometric thresholds were compared with those of a control group of children (*n* = 57) from the same area. *Findings*. During the acute stage, hearing threshold levels of treated children were significantly elevated compared with controls (*P* < 0.001). The threshold elevations persisted up to 28 days, but no differences in hearing thresholds were evident between treated children and controls after 9 months. The hearing thresholds of children treated with the two ACT regimens were comparable but lower than those of amodiaquine-treated children during acute illness. *Interpretation*. Malaria is the likely cause of the elevated hearing threshold levels during the acute illness, a finding that has implications for learning and development in areas of intense transmission, as well as for evaluating potential ototoxicity of new antimalarial drugs.

## 1. Background

Acute *Plasmodium falciparum* malaria is associated with varying degrees of neurological involvement, depending on the severity. Few studies have, however, investigated the effect of the disease on hearing specifically in those with uncomplicated malaria. Furthermore, several antimalarial drugs, including quinine [1, 2], chloroquine [3], and mefloquine [4], have been associated with ototoxicity, and certain artemisinin derivatives have also been associated with neuro- or ototoxicity in various animal species [5–9]. Although human studies that have evaluated possible artemisinin-related effects on hearing have, with the exception of one study [10], concluded lack of any clinically relevant ototoxicity or neurotoxicity [11–15], the majority of these studies have been done in adults, in spite of the fact that children are the subgroup of patients who, because of their still developing nervous systems, are more susceptible to such potential treatment-related effects. The lack of studies evaluating the potential effects of newly introduced antimalarial drugs on hearing in children is due to the difficulties in conducting serial audiometric measurements in childhood populations, especially in resource-poor malaria-endemic countries where the disease is most prevalent. In most malaria-endemic countries, there is, indeed, near-absence of pre-illness hearing threshold data with which to compare data obtained during acute illness. This makes it difficult to conduct hearing assessment studies during malarial illness, as it becomes nearly impossible to distinguish between disease-specific and emergent or drug-related effects in such areas. In spite of these challenges, however, studies investigating trends in hearing thresholds during malaria are important, because malaria, even when uncomplicated, has been implicated as a cause of elevated audiometric thresholds in experimentally infected (nonimmune) adults with uncomplicated malaria [14], as well as in animals [16]. In addition, repeated attacks of uncomplicated malaria have been implicated as a cause of poor cognitive performance in school children [17], but few studies have focused audiometric studies specifically on children.

We have, in this study, conducted serial audiometric measurements with a follow-up time ranging between 9 and 15 months, in children with uncomplicated malaria treated with artesunate-amodiaquine, artemether-lumefantrine, or amodiaquine monotherapy. These measurements have been done not only to evaluate artemisinin-based and non-artemisinin-based antimalarial treatments, but also to compare artemisinin combination therapy (ACT) regimens that have been associated with differential propensities for neurotoxicity in animal studies. The audiometric measurements were also compared with those of age-matched children from the same area. The study design also permits comparison of audiograms obtained during acute illness with audiograms done at different postrecovery time points, allowing assessment of the potential effect of the acute malarial disease and recovery on the dynamics of hearing threshold changes over the study period.

## 2. Materials and Methods

### 2.1. Study Site and Subjects

The audiometric assessment was a subcomponent of a clinical trial that was done to evaluate the efficacy and safety of artemisinin-based combination therapies for uncomplicated malaria in Ghana. Approval for the study was granted by the Ethics and Protocol Review Committee of the University of Ghana Medical School. The clinical trial was initiated at a time when chloroquine was still the official first-line treatment for uncomplicated malaria in Ghana. The full description of the study site and trial results have been previously reported [18]. Briefly, enrolled children aged 0.5–14 years with uncomplicated malaria were treated with (i) artesunate (Plasmotrim, Mepha; Switzerland), 4 mg/kg body weight as a single daily dose + amodiaquine (Camoquine Pfizer; Dakar, Senegal), 10 mg/kg body weight single daily dose, for 3 days, or (ii) artemether-lumefantrine (Coartem, Novartis Pharma AG, Basel, Switzerland; 20 mg artemether and 120 mg lumefantrine), given at 0 and 8 hours on the first day and then twice daily for the two subsequent days according to body weight: 9–14 kg, 1 tablet/dose; 15–24 kg, 2 tablets/dose; 25–34 kg, 3 tablets/dose; 35 kg and over, 4 tablets/dose. A limited number of children were treated with amodiaquine monotherapy (same dosage as above); however, this (monotherapy) treatment arm was discontinued in early 2005 when the Ghana national first-line antimalarial treatment policy was changed from chloroquine to artesunate-amodiaquine. This monotherapy-treated-group, however, is crucial for inclusion as non-artemisinin treated control subjects in the context of the objectives of this study and has been maintained in the data analysis despite its limited size. In addition, a control group of randomly selected age- and sex-matched children were enrolled from a school in the study area as controls. 

### 2.2. Study Procedures and Treatments

Otoscopic examination was done in all children as a part of the initial screening. Children with conditions such as serous otitis media, active or inactive chronic suppurative otitis media, impacted cerumen, eardrum perforation, tympanic membrane scarring, or other clinically evident outer and middle ear abnormalities, were excluded. Children with a past medical history or clinically obvious symptoms and signs of sickle cell anaemia, renal or liver disease, malnutrition, craniofacial abnormalities, or dysmorphism, as well as children, when they were newborns, were reported to have been admitted into neonatal intensive care for any cause or those with a history of head trauma were excluded. Additionally, children with a past medical history suggesting possible birth asphyxia, neonatal jaundice, and meningitis, and those who were known to have taken aminoglycoside antibiotics, loop diuretics, or herbal medications within the past three months were excluded.

### 2.3. Audiometry

Air conduction threshold was obtained for each ear separately in a quiet (nonairtight) room, using a portable audiometer (Kamplex KS8; PC Werth, London, UK), with noise-attenuated TDH-39 headsets and earphones. The audiometer was calibrated according to the manufacturer's recommendations by an external expert. The level of ambient noise measured in the examination room was <50 dB A (within the level recommended by the American National Standard for ambient noise levels for audiometric test room standards). The unmasked psychoacoustical hearing thresholds were established at 0.125, 0.25, 0.5, 0.75, 1, 1.5, 2, 3, 4, 6, and 8 kHz, for tonal stimuli, using a standard two-step-down, one-step-up method, as per the modified Hughson-Westlake procedure [19]. Briefly, pure tones at the specified frequency were applied and then decreased, in 10 dB steps until no response was elicited from the subject. The applied tone was subsequently increased in 5 dB steps until a response elicited. This procedure was repeated, and the hearing threshold at a particular frequency was determined as the average of the lowest sound intensity the subject responded to. The control subjects were tested once, under similar conditions, using the same equipment, and following the same procedure as the subjects. 

### 2.4. Laboratory Investigations

Venous blood was collected into EDTA and heparinised tubes on days 0, 3, 7, 14, and 28 and on any day of recurrent symptomatic parasitaemia for routine haematological and biochemical investigations. Parasite counts were determined in Giemsa-stained blood films relative to 200 white blood cells (WBCs) and the measured WBC count. Total WBCs and differential counts were measured by an automated haematology analyzer (CELL DYN, Abbott Laboratories, USA).

### 2.5. Statistical Analysis

Continuous normally distributed data were described by the mean and standard deviation or standard error and non normally distributed data by the median and range. For the latter data, all statistics were performed on ranks. Percentages were given for categorical data, which were compared using the Chi-square test with Yates' correction, or Fischer's exact test, as appropriate. One-way analysis of variance, with Holm-Sidak post hoc pairwise testing, or two-way repeated measures analysis of variance was used to compare variables between the treatment groups and controls or to test for intra-individual and within-group differences, respectively. For some comparisons, a delta-value was calculated by subtracting the values measured at two time points in the same individual. Multiple linear regression analysis was used to evaluate the effect of selected subject characteristics on the audiometric thresholds. *P* values <0.05 were considered significant. Data are presented for selected wavelengths for clarity. Inclusion of additional wavelengths in the analysis did not alter the conclusions. Statistics were done, using SigmaPlot 11 (Systat, Chicago, IL, USA).

## 3. Results

Audiometric analysis was performed in 80 malaria patients (artesunate-amodiaquine, *n* = 37; artemether-lumefantrine, *n* = 35; amodiaquine, *n* = 8) on days 0, 3, 7, and 28; however, for each day of repeated testing, various children did not turn up, explaining the variable numbers of comparison in each analysis below. In all repeated comparisons, only patients tested at both time points were included in the data analysis. At the end of the follow-up period, audiometry was done in 58 of the originally recruited 80 treated children (artesunate-amodiaquine, *n* = 30; artemether-lumefantrine, *n* = 23; amodiaquine, *n* = 5) and in 57 healthy controls. 

### 3.1. Hearing Threshold Levels on Admission (before Treatment)

**Figure 1:**
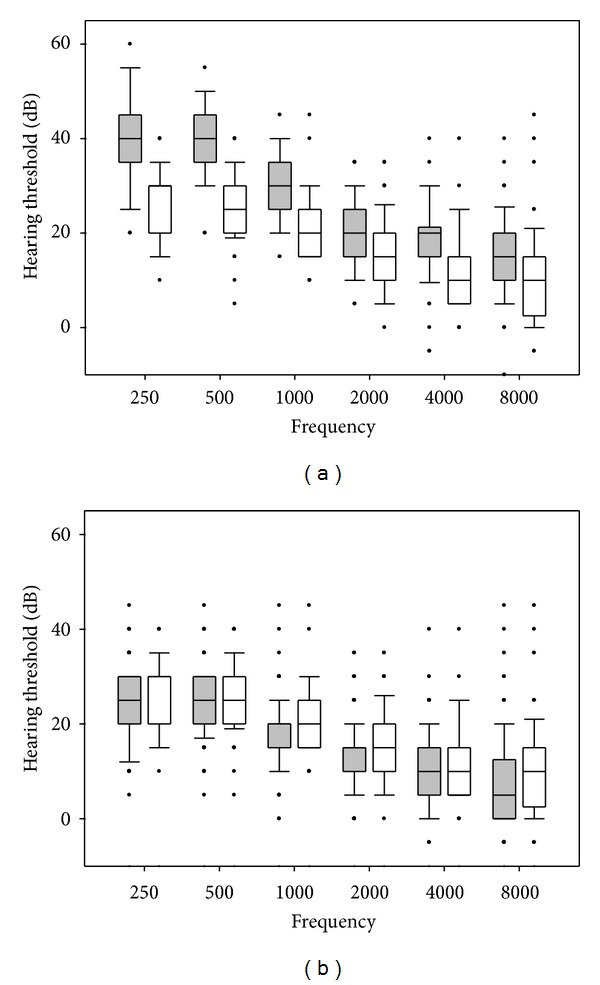
(a) Audiometry data on 80 patients with uncomplicated malaria before treatment (shaded) and 57 healthy controls (open). Box plots show mean, quartiles, 95% and 5% percentiles, and outliers. Data for right ear have been shown in all figures; measurements on the left ear gave essentially identical results throughout. Hearing thresholds were significantly higher in patients than in controls across all wavelengths (two-way repeated measurement ANOVA on ranks *P* < 0.001, post hoc test significant (*P* < 0.05) for all wavelengths). (b) Audiometry data on 58 patients followed up 9–12 months after an attack of uncomplicated malaria (shaded) and 57 healthy controls (open, same as in (a)). Hearing thresholds were significantly lower in recovered patients than controls across all wavelengths and both ears (two-way repeated measurement ANOVA on ranks *P* = 0.01, median difference <5 dB).

On day 0 (before treatment), hearing thresholds of all the acutely ill subjects were significantly elevated compared with the hearing thresholds of the control subjects (Figure 1(a), two-way repeated measurement ANOVA on ranks *P* < 0.001, post hoc test significant for all frequencies). Hearing threshold levels were similar for the three treatment groups, each of which was significantly different from the control group (except 6000 Hz, Table 1).

### 3.2. Changes in Hearing Thresholds from Day 0 to Day 28

**Figure 2:**
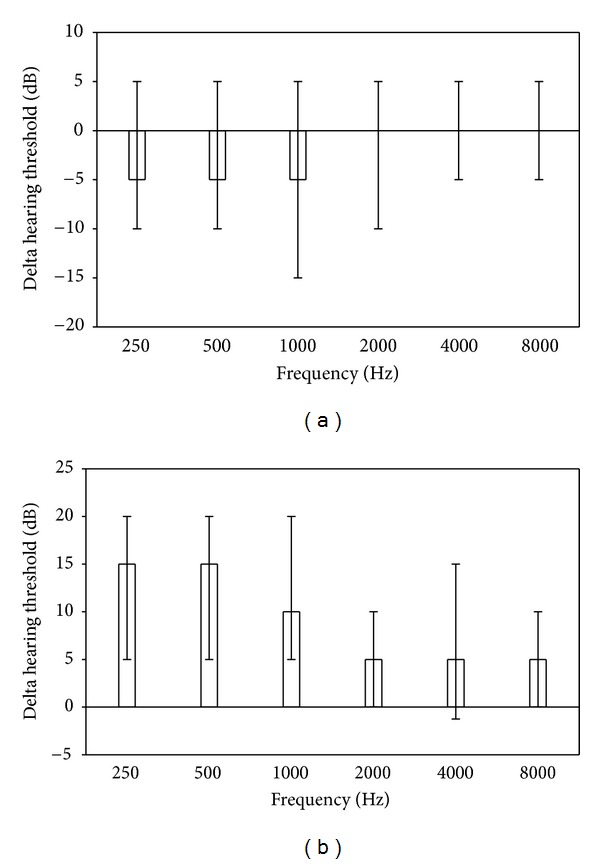
(a) Changes in hearing threshold 28 days after start of treatment of uncomplicated malaria relative to threshold before treatment (median, 25% and 75% quartiles). Hearing improvement compared with day 0 was significant at wavelengths 250 Hz and 500 Hz and for left ear also 1000 Hz (signed rank test performed for each frequency and ear, *P* < 0.05, *n* = 47). (b) Changes in hearing threshold 28 days after start of treatment of uncomplicated malaria relative to threshold at follow-up 9–12 months later. Hearing was significantly impaired on day 28 as compared with followup at all wavelengths and both ears (signed rank test, *P* < 0.01, *n* = 38).

There were no differences in hearing threshold levels from day 0 to day 3 or from day 3 to day 7 (*P* between 0.1 and 0.9, signed rank test for all frequencies/ears and both days, data not shown). There were significant improvements (decreases) in hearing threshold levels on day 28 compared with day 0, at specific frequencies (250 Hz and 500 Hz for both ears and also at 1000 Hz for the left ear, signed rank test performed for each frequency and ear, *P* < 0.05, *n* = 47, Figure 2(a), for a representation across all wavelengths). Of a particular note, despite the improvement, the mean hearing threshold levels remained significantly elevated on day 28 compared with followup at 9–15 months (Figure 2(b), *P* < 0.01, *n* = 38).

### 3.3. Hearing Threshold Levels after 9–15 Months

The hearing threshold level measurements of treated children measured after at least 9 months were comparable, or even marginally (but clinically nonsignificantly), lower than the hearing thresholds of controls (Figure 1(b), two-way repeated measurement ANOVA on ranks *P* = 0.01, median difference <5 dB), except for measurements for the right ear at 125 Hz and 250 Hz (Table 2).

### 3.4. Comparison of Hearing Thresholds between Treatment Groups

**Figure 3:**
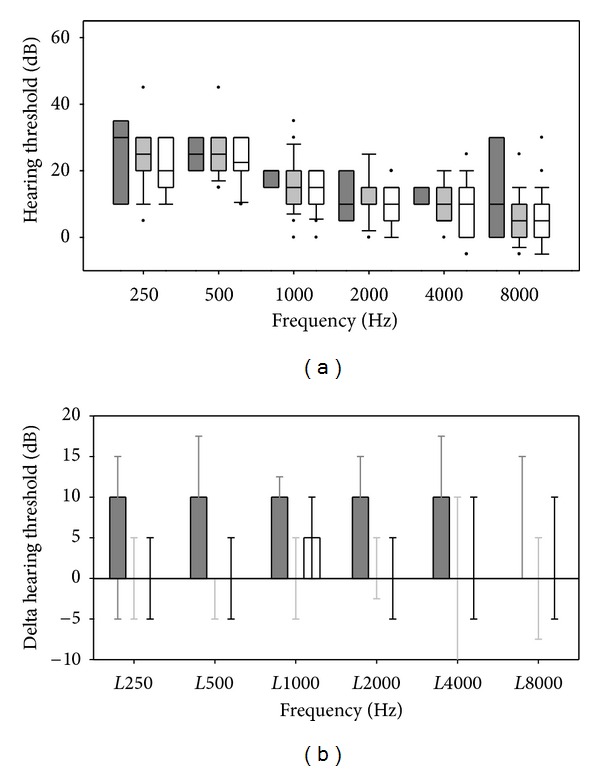
(a) Audiometry data at 9–12 months followup by treatment group. Amodiaquine (dark shaded, *n* = 3), artemisinin-lumefantrine (light shaded, *n* = 23), artesunate-amodiaquine (open, *n* = 30). No difference could be shown across all wavelengths and both ears (two-way repeated measurement ANOVA on ranks *P* = 0.7). (b) Hearing threshold on day 7 after treatment relative to threshold before treatment by group. Colours as (a). Columns and error bars as in Figure 2. Patients treated with amodiaquine alone had significant increase in hearing threshold across all wavelengths compared with patients treated with ACT, who had unchanged threshold compared to day 0 (two-way repeated measurement ANOVA on ranks *P* < 0.001, Holm-Sidak post hoc test of amodiaquine versus artemether-lumefantrine *P* < 0.001, of amodiaquine versus artesunate-amodiaquine *P* = 0.003, amodiaquine *n* = 5, artemether-lumefantrine *n* = 21, artesunate-amodiaquine *n* = 15).

At the final followup (9–15 months), there were no differences in the mean hearing threshold levels between the amodiaquine monotherapy and the artesunate-amodiaquine or artemether-lumefantrine arms (Figure 3(a), two-way repeated measurement ANOVA on ranks, *P* = 0.7). On day 7, the hearing threshold levels in the amodiaquine monotherapy arm were higher, whereas in the two ACT arms they remained at the level of day 0. Thus, the hearing threshold levels on day 7 in the monotherapy arm were significantly higher than the ACT arms, especially at frequencies <1000 Hz (Figure 3(b), two-way repeated measurement ANOVA on ranks, *P* < 0.001, Holm-Sidak post hoc test for amodiaquine versus artemether-lumefantrine, *P* < 0.001, of amodiaquine versus artesunate-amodiaquine, *P* = 0.003; amodiaquine, *n* = 5, artemether-lumefantrine, *n* = 21, artesunate-amodiaquine, *n* = 15).

### 3.5. Clinical and Demographic Characteristics and Hearing Threshold Levels

A linear regression analysis performed to compare the effect of selected characteristics on hearing threshold levels showed that a lower mean age and disease severity (i.e., higher parasite density) were the most consistent predictors of elevated hearing threshold measured on the exit (follow-up) audiogram (data not shown).

## 4. Discussion

In this study, serial audiometric measurements were done before treatment and at prespecified times after treatment of uncomplicated malaria, with ACT regimens that have been associated with different propensities for toxicity in animal studies, as well as in subjects treated with a nonartemisinin (amodiaquine) antimalarial drug. The results showed elevated hearing thresholds in all three groups of children at presentation and through the acute illness stage but complete reversibility after a sufficiently long follow-up period. Although the data shows progressive improvement (decrease) of hearing threshold levels during the initial days of treatment, especially in the two ACT groups, there were some residual elevation 28 days after treatment. The pattern of hearing threshold elevations, which was more pronounced at the lower frequencies, is consistent with that of the malaria-attributed hearing impairment that has been recently demonstrated in animals [16].

It has also been shown, by fundoscopic examinations, that uncomplicated malaria may be associated with some neurological effects [20], a finding that has potential implications for development, especially in areas of intense malaria transmission. In this respect, the trend towards widening the target group for intermittent preventive treatment for malaria with the aim of reducing the number of malaria attacks in certain settings [21, 22] could be partly supported by the findings from this study.

The data did not show any differences between hearing threshold levels of children treated with artesunate-amodiaquine or artemether-lumefantrine. However, treatment with ACT-based antimalarials seemed to halt the persistence or progression of the threshold elevations during acute illness, whereas amodiaquine monotherapy treatment did not show such an effect. Although the number of subjects in the amodiaquine monotherapy group was limited, precluding definitive conclusions on differences between ACT and non-ACT treatment groups, this apparent difference between the ACT and non-ACT antimalarial regimens could be due to the rapid parasite clearance and faster fever resolution in the ACT groups [18]. The quicker symptom resolution in the ACT groups would be expected to have relatively lower effect on subject concentration and ability to respond to the tonal stimuli. However, the artemisinin derivatives may also play a direct role by modifying cerebral immunopathology, as described in a murine model of experimental cerebral malaria [23].

The findings of this study therefore suggest that there were minimal, if any, detectable effect of the two ACT regimens, at the administered doses, on hearing as measured by audiometry. Furthermore, since hearing threshold change is considered a better indicator of potential drug-induced ototoxicity than hearing threshold levels [24], the complete reversibility of the noted changes further suggest disease- rather than drug-induced changes. However, a more definitive statement of a lack of effect of treatment with these ACT regimens on hearing threshold changes could have been made if plasma concentration data of dihydroartemisinin, the main metabolite of artemisinins, were available. Also, since preillness baseline audiometric threshold levels were not available in this study—as in most malaria audiometric studies—further studies that utilize preillness audiometric thresholds as a baseline should be considered—in so far as this is feasible in endemic areas. The treatment responses from the efficacy trial, however, suggest that therapeutic levels were attained.

The complete reversibility of the elevated threshold elevations in all the three groups at the nine-month follow-up audiogram suggests acute malaria to be the likely cause of these changes, which is consistent with results from studies in adults [12, 13, 15]. Furthermore, the (slightly) better performance of subjects compared with controls at the exit audiogram is possibly due to a learning effect, most likely from repeated testing, as it has been reported that test scores of pure tone audiometry in children, improve with age or after repeated examinations [25–27].

It has been hypothesized that malaria could contribute to hearing impairment by impairing labyrinth artery microcirculation [28] and it has also been shown that antimalarial drugs could, by disrupting cochlear vasculature, increase susceptibility to ototoxicity [29].

These findings taken together imply that not only does hearing impairment occur as part of the natural history of uncomplicated malaria, but also if audiometry is used to evaluate drug induced ototoxicity of newly introduced antimalarial drugs, the effect of the disease could confound any hearing threshold elevations if testing of different individuals is done at different posttreatment times.

## 5. Conclusion

Audiometric thresholds measured in children with uncomplicated malaria treated with different antimalarial regimens showed reversible hearing threshold elevations in all treated groups, implying that these changes were disease- rather than drug-related. This has potential implications for learning, development, and behaviour of children repeatedly exposed to malaria in endemic areas and also has implications for evaluating potential ototoxicity of newly introduced antimalarial drugs. 

## Figures and Tables

**Table 1:** Median hearing threshold levels on day 0 (before treatment).

Frequency	Group	Left ear			Right ear		
		Median	IQR	*P*-value	Median	IQR	*P*-value
125	ASAQ	30	27.5–35	0.001	30	25–40	0.001
	AML	40	30–40		40	30–40	
	AQ	35	35–37.5		40	30–42.5	
	CTRL	25	20–30		25	20–30	

250	ASAQ	40	35–45	0.001	35	35–41.25	0.001
	AML	42.5	37.5–45		45	32.5–50	
	AQ	45	43.75–45		40	36.25–46.25	
	CTRL	25	20–30		30	20–30	

500	ASAQ	40	35–40	0.001	40	35–41.25	0.001
	AML	40	37.5–50		40	32.7–47.5	
	AQ	45	38.75–45		45	42.5–46.25	
	CTRL	25	20–30		25	20–30	

750	ASAQ	35	30–40	0.001	30	30–40	0.001
	AML	40	35–45		37.5	30–40	
	AQ	40	33.75–41.25		40	36.25–45	
	CTRL	25	20–30		25	20–26.25	

1000	ASAQ	30	20–30	0.001	25	22.5–32.5	0.001
	AML	32.5	30–35		30	27.5–35	
	AQ	35	30–35		35	26.25–36.25	
	CTRL	20	15–25		20	15–25	

1500	ASAQ	25	25–30	0.001	20	20–28.75	0.001
	AML	30	25–30		30	21.25–20	
	AQ	25	23.75–31.25		27.5	20–30	
	CTRL	15	13.75–20		15	15–21.25	

2000	ASAQ	20	15–20	0.001	20	15–20	0.001
	AML	20	20–30		20	17.5–27.5	
	AQ	20	20–25		20	12.5–26.25	
	CTRL	15	10–20		15	10–20	

3000	ASAQ	20	10–21.25	0.001	15	13.75–20	0.001
	AML	20	15–25		20	15–27.5	
	AQ	20	15–22.5		15	13.75–20	
	CTRL	15	8.75–15		10	7.5–15	

4000	ASAQ	15	10–21.25	0.001	15	15–20	0.001
	AML	20	15–25		20	15–25	
	AQ	15	15–21.25		15	13.75–16.25	
	CTRL	10	10–16.25		10	5–15	

6000	ASAQ	20	13.75–25	0.09	15	10–26.5	0.13
	AML	22.5	20–30		20	10–30	
	AQ	20	13.75–21.25		15	6.25–21.25	
	CTRL	15	10–20		15	10–20	

8000	ASAQ	10	5–16.25	0.02	15	10–15	0.02
	AML	15	10–25		15	10–20	
	AQ	20	13.75–28.75		15	5–20	
	CTRL	10	5–15		10	3.75–15	

ASAQ = artesunate + amodiaquine; AML: artemether-lumefantrine; AQ: amodiaquine; CTRL: control group.

**Table 2:** Median hearing threshold levels after 9–12 months.

Frequency	Drug	Left ear			Right ear		
		Median	IQR	*P*-value	Median	IQR	*P*-value
125	ASAQ	22.5	20–30	0.47	20	10–25	0.03
	AML	20	16.25–25		20	16.25–25	
	AQ	25	21.25–28.75		25	17.50–28.75	
	CTRL	25	20–30		25	20–30	

250	ASAQ	25	20–30	0.39	20	15–30	0.04
	AML	25	20–30		25	20–30	
	AQ	35	27.5–35		30	15–33.75	
	CTRL	25	20–30		30	20–30	

500	ASAQ	25	20–30	0.78	22.5	20–30	0.10
	AML	25	20–30		25	20–30	
	AQ	30	18.75–33.75		25	21.25–28.75	
	CTRL	25	20–30		25	20–30	

750	ASAQ	20	20–25	0.792	20	15–20	0.14
	AML	25	20–25		20	20–25	
	AQ	25	17.5–28.75		25	17.5–28.75	
	CTRL	25	20–30		25	20–26.25	

1000	ASAQ	17.5	15–20	0.67	15	10–20	0.06
	AML	20	15–20		15	11.25–20	
	AQ	20	16.25–20		20	16.25–20	
	CTRL	20	15–25		20	15–25	

1500	ASAQ	15	10–20	0.88	15	10–20	0.25
	AML	15	15–20		15	11.25–20	
	AQ	15	15–18.75		15	11.25–18.75	
	CTRL	15	13.75–20		15	15–21.25	

2000	ASAQ	15	10–20	0.82	10	5–15	0.03
	AML	10	10–15		10	10–15	
	AQ	15	11.25–15		10	6.25–17.50	
	CTRL	15	10–20		15	10–20	

3000	ASAQ	10	5–15	0.60	10	5–10	0.06
	AML	10	6.25–15		10	6.25–15	
	AQ	15	11.25–15		5	5–12.5	
	CTRL	15	8.75–15		10	7.5–15	

4000	ASAQ	10	5–20	0.65	10	0–15	0.23
	AML	10	6.25–18.75		10	5–15	
	AQ	15	3.75–15		10	10–13.75	
	CTRL	10	10–16.25		10	5–15	

6000	ASAQ	10	10–25	0.23	10	5–15	0.18
	AML	15	15–25		10	5–15	
	AQ	15	11.25–22.5		10	10–13.75	
	CTRL	15	10–20		15	10–20	

8000	ASAQ	10	5–10	0.70	5	0–10	0.13
	AML	10	5–15		5	0–10	
	AQ	5	1.25–16.25		10	2.5–25	
	CTRL	10	5–15		10	3.75–15	

ASAQ = artesunate + amodiaquine; AML: artemether-lumefantrine; AQ: amodiaquine; CTRL: control.
